# Genetic Variants Associated with Biological Treatment Response in Inflammatory Bowel Disease: A Systematic Review

**DOI:** 10.3390/ijms25073717

**Published:** 2024-03-27

**Authors:** Javier Plaza, Alejandro Mínguez, Guillermo Bastida, Remedios Marqués, Pilar Nos, Jose Luis Poveda, Inés Moret-Tatay

**Affiliations:** 1 Inflammatory Bowel Disease Research Group, Health Research Institute La Fe (IIS La Fe), 46026 Valencia, Spain; javier.plaza.soriano@gmail.com (J.P.); alejandromsab11@gmail.com (A.M.); 2 Department of Pharmacy and Pharmaceutical Technology and Parasitology, University of Valencia, 46100 Valencia, Spain; 3 Inflammatory Bowel Disease Unit, Gastroenterology Department, La Fe University and Polytechnic Hospital, 46026 Valencia, Spain; bastida_gui@gva.es (G.B.); nos_pil@gva.es (P.N.); 4 Pharmacy Department, La Fe University and Polytechnic Hospital, 46026 Valencia, Spain; marques_mre@gva.es (R.M.); poveda_josand@gva.es (J.L.P.); 5 General Directorate of Public Health, Council of Healthcare, 46021 Valencia, Spain

**Keywords:** inflammatory bowel disease, biological drugs, anti-tumor necrosis factor, infliximab, adalimumab, ustekinumab, ulcerative colitis, Crohn’s disease

## Abstract

Inflammatory bowel disease (IBD) is a chronic inflammatory disorder of the digestive tract usually characterized by diarrhea, rectal bleeding, and abdominal pain. IBD includes Crohn’s disease and ulcerative colitis as the main entities. IBD is a debilitating condition that can lead to life-threatening complications, involving possible malignancy and surgery. The available therapies aim to achieve long-term remission and prevent disease progression. Biologics are bioengineered therapeutic drugs that mainly target proteins. Although they have revolutionized the treatment of IBD, their potential therapeutic benefits are limited due to large interindividual variability in clinical response in terms of efficacy and toxicity, resulting in high rates of long-term therapeutic failure. It is therefore important to find biomarkers that provide tailor-made treatment strategies that allow for patient stratification to maximize treatment benefits and minimize adverse events. Pharmacogenetics has the potential to optimize biologics selection in IBD by identifying genetic variants, specifically single nucleotide polymorphisms (SNPs), which are the underlying factors associated with an individual’s drug response. This review analyzes the current knowledge of genetic variants associated with biological agent response (infliximab, adalimumab, ustekinumab, and vedolizumab) in IBD. An online literature search in various databases was conducted. After applying the inclusion and exclusion criteria, 28 reports from the 1685 results were employed for the review. The most significant SNPs potentially useful as predictive biomarkers of treatment response are linked to immunity, cytokine production, and immunorecognition.

## 1. Introduction

Inflammatory bowel disease (IBD) is a complex and multifactorial relapsing gastrointestinal inflammatory condition with a continuously increasing incidence and can involve the entire gastrointestinal tract [1,2]. Crohn’s disease (CD) and ulcerative colitis (UC), the two main types of IBD, affect up to 1% of the population, negatively influencing the quality of life of patients and their relatives [3,4]. The location of the inflammation, the disease’s behavior, the symptoms, and the nature of the histological gastrointestinal tract disorders differentiate between the two diseases. The interplay between several factors, such as the host’s intestinal microbiota, immune system, genetic predisposition, and environmental factors (e.g., drugs, diet, smoking) affect the onset and development of IBD. However, the precise cause of the disease remains unknown [1,4].

Several proinflammatory cytokines, such as interleukin (IL)-17, IL-6, interferon-C, and tumor necrosis factor (TNF)-α, are secreted after the triggering of various pathways as a result of the stimulation of Toll-like receptors and NOD-like receptors on epithelial cells and local immune cells by microbes of the microbiota [4,5,6,7,8,9,10]. This process results in impaired microbe sensing (mainly in CD), disturbance of the barrier function (mainly in UC), and abnormal regulation of adaptive immune responses (in both diseases) [4,9]. Specifically, increased TNF-α expression might decrease the mucosal barrier function due to TNF signaling, which influences the apoptosis of intestinal epithelial cells and induces changes in the epithelial expression of tight junction proteins via caspase-8 activation. Furthermore, the biological activity of TNF, mediated by its binding to TNF receptor type 1 (TNFR1) and type 2 (TNFR2), is crucial for cell proliferation, differentiation, and pro-inflammatory signaling [4,5,6,7,8,9,10].

Biologics are bioengineered therapeutic drugs that target a gene or protein, primarily cytokines. This pharmacologic option, employed in many autoimmune diseases, has also been applied to IBD therapy to reduce hospitalization and surgical rates [11]. Biologics focus on TNF blockade, and anti-TNF agents decrease the downstream effects of TNF activation. Infliximab (IFX) and adalimumab (ADA) are the two classical main anti-TNF drugs employed in routine clinical practice [12,13,14].

In addition to anti-TNF agents, other novel biological agents have been implemented in IBD therapy, such as ustekinumab (UST) and vedolizumab (VDZ), which work against anti-IL12/23 and anti-α4β7 integrin, respectively [15,16]. UST is a monoclonal antibody that neutralizes IL-12 and IL-23 by targeting the shared p40 subunit and is effective in treating patients with CD after failure of targeted TNF-α therapy [17,18,19,20]. Given that IL-23 targets an important agent involved in the development of autoinflammatory subsets of helper T cells (Th17), UST efficacy relies on this interleukin rather than IL-12 [17,20,21]. VDZ is a humanized monoclonal antibody that targets the α4β7 heterodimer, a surface protein present in gut-specific lymphocytes. Due to this binding, VDZ works by reducing the flow of lymphocytes to the gut [22,23].

**Figure 1:**
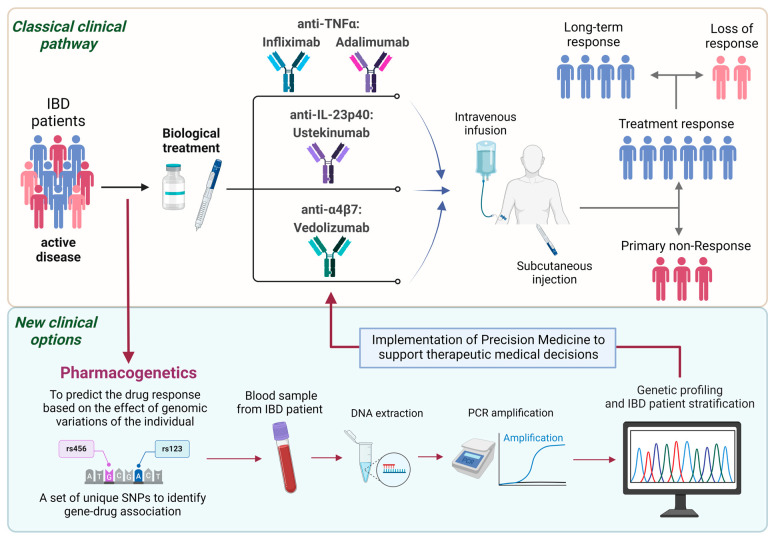
An overview of the use of pharmacogenetics in precision medicine in inflammatory bowel disease to predict drug response and tailor the therapy to the patient’s needs. Created by Inés Moret-Tatay with BioRender.

Despite the beneficial therapeutic options of biologics in IBD (Figure 1), patients can develop persistent symptoms and disease activity [24]. Between 10% and 40% of patients with IBD do not respond to the remission induction regimen (known as non-response or failure) and, among primary response patients, up to 30–40% will lose the response during anti-TNF maintenance therapy (secondary non-response or secondary failure) [20,21,25,26]. In the long term, only one-third of the patients will therefore eventually respond to these drugs in the blood. The biological drug’s immunogenicity (which results in low blood levels of the active therapy), pharmacokinetics [13,14], and inflammatory burden appear to play a role in the reduced pharmacological effect [25,26].

Personalized medicine has therefore emerged as a useful tool for predicting responsiveness to biological agents in patients with IBD (Figure 1) [27,28]. Younger age, short disease duration, and luminal disease have been associated with a beneficial response. C-reactive protein (CRP) levels, fecal calprotectin, and serological markers might also have predictive value [29,30]. Current data suggest that patients with IBD who carry specific gene alleles are at high risk of low anti-TNF blood concentrations and can develop immunogenicity to these drugs, as observed in the prospective study Personalized Anti-TNF Therapy in Crohn’s Disease (PANTS) [31]. Studying genetic variants can therefore help identify underlying factors affecting the clinical response to biologics. Pharmacogenetic studies might be useful for classifying a subset of patients who are likely to present a beneficial response to a biological agent (Figure 1). In general, single nucleotide polymorphisms (SNPs) in the pharmacokinetic and pharmacodynamic pathways can be relevant in predicting therapeutic efficacy. Moreover, novel therapeutic strategies could be developed based on the genetic profiles of non-responders [32,33,34].

Here, we present a review of the current knowledge on genetic variants associated with biological agent treatment response in patients with IBD. Our aim has been to identify potential candidate SNPs based on the available genomic data as predictive biomarkers of response among patients with IBD, thereby minimizing adverse effects and more optimally employing healthcare resources.

## 2. Materials and Methods

### 2.1. Information Sources and Search, Study Selection

**Figure 2:**
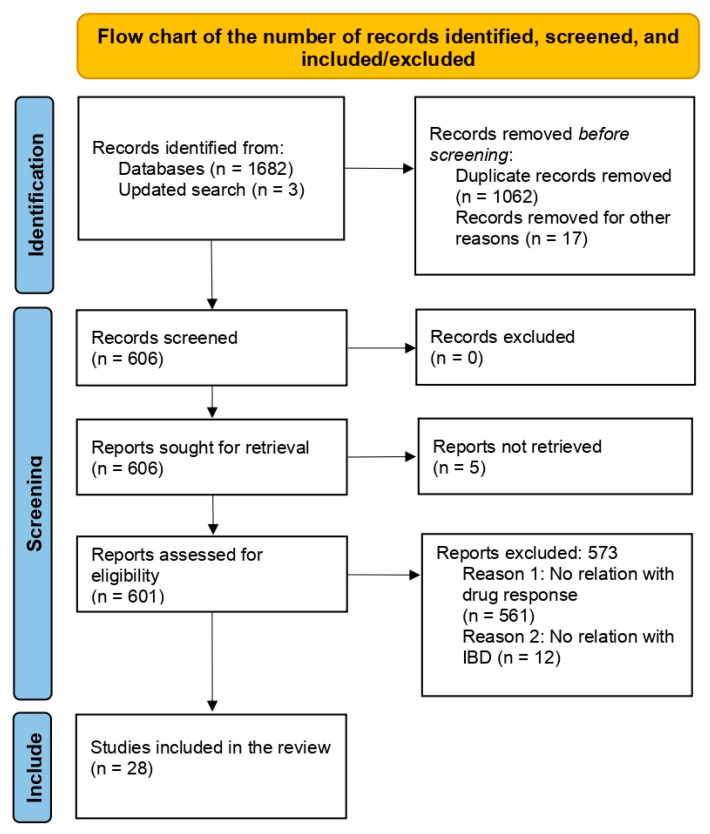
Flow chart for studies included in this review. First, we identified the publications via an online literature search. Using manual screening, we then excluded publication types not reporting new measurements/data, as well as repeated studies. The remaining publications were assessed for eligibility. Those reporting suitable (quantifiable) data on genomic or expression markers of biological drug response measured prior to the start of therapy were included in the review.

An online literature search of PubMed and Web of Science was performed (latest search date: 8 January 2024; Supplementary Table S1 shows the search strategy) from studies suggesting that they presented original data on polymorphisms and biological treatment response. The complete search process is illustrated in the PRISMA (Preferred Reporting Items for Systematic Reviews) flow chart (Figure 2). We excluded 1657 studies due to missing data, duplicates, or no association with drug response, and studies based on the pharmacogenetics of other immunomodulators for IBD, such as azathioprine and methotrexate. Ultimately, we included 28 studies that reported on genetic markers and anti-TNF response in IBD. No further studies were identified by searching the literature list of the retrieved articles. Data from articles with available data on odds ratios (ORs) and 95% confidence intervals (CIs) or number of responders (complete or partial), non-responders, and genotypes were included. In addition, we searched the resource database PharmGKB “https://www.pharmgkb.org/” (accessed on 21 November 2023) to obtain further information on the identified genetic variants and the IBD biological treatment relationship.

The reports were revised independently by three separate reviewers to select those that met the review’s inclusion criteria.

### 2.2. Data Collection and Integration

We collected data on genomic biomarkers measured in the peripheral blood mononuclear cells of patients with IBD, including all the relevant data, regardless of the therapy employed (IFX, ADA, UST) or how the clinical response was assessed (e.g., Crohn’s Disease Activity Index [CDAI], Harvey–Bradshaw Index [HBI], Inflammatory Bowel Disease Questionnaire [IBDQ], clinical assessment). For genomic markers, we collected data on all SNPs that were associated with biological therapy response with a *p*-value < 0.05.

### 2.3. Evaluation of the Drug Response

The therapeutic response was assessed differently between the studies included in this review. In the pediatric population, the Pediatric Ulcerative Colitis Activity Index (for UC) and the Weighted Pediatric Crohn’s Disease Activity Index (for CD) were employed. In the adult population, the simple 3-step clinical scale (no response, partial response, response), clinical activity indices such as the CDAI and HBI (CD), and quality-of-life indexes such as the IBDQ were employed, in addition to biochemical parameters such as CRP. In perianal disease, the response was evaluated according to the decrease in the fistula output and/or by their closure. The persistence of the drug or its suspension/modification was also employed as response/non-response criteria (Supplementary Table S2). Other studies did not detail the criteria employed.

Another discrepancy in the studies was the drug response evaluation time. Although a number of studies assessed the response immediately after drug induction (weeks 10–12), other studies had a delayed time of up to 6 months (approximately weeks 20–30) or 1 year (week 52). To avoid fluctuations, other studies employed the means of clinical indices and biochemical parameters during the first year of therapy.

## 3. Results

### 3.1. Literature Search

The electronic literature search yielded 1685 results (Figure 2), obtained as follows: any results with missing data, repeated articles, or no association with drug response were excluded. The remaining 606 publications were assessed for eligibility. The publications not reporting on genomic or expression biomarkers, not comparing non-responders with responders, or reporting only expression data after the start of therapy were excluded. The remaining 28 publications (Supplementary Table S1) were included in the systematic review.

### 3.2. Polymorphisms Associated with Responsiveness among Patients with Inflammatory Bowel Disease Treated with Infliximab

The information collected from the studies is summarized in Table 1. As indicated, 5 studies were identified that associated SNPs with responsiveness to IFX in patients with IBD. It should be noted that most of the studies had been developed in cohorts of White patients (the majority of adults).

A number of the analyzed polymorphisms were related to beneficial outcomes in the long-term response to IFX therapy in patients with IBD. The most investigated SNPs are related to pathogen recognition: *TLR2* (rs1816702 CC and rs3804099 TT) [35,36]. In an Italian cohort of 76 pediatric patients with IBD, only the SNP rs396991 GG of gene *FCGR3A* was linked to IFX non-response [37].

Within short-term responses, no SNPs were linked to a beneficial response. In fact, the studies showed that all the SNPs related to short-term response were associated with non-response to IFX. Those SNPs have been identified in genes related to the TNF-α pathway, *TNFRSF1B* (rs976881 AA + GA) [38], and the immunoglobulin superfamily (nervous system development) *CNTN5* (rs1813443 CC and rs1568885 TT) in patients with CD [39].

The SNP rs1061624 (AA + GA) of the *TNFRSF1B* gene presents controversial outcomes. One study [35] observed that this polymorphism was related to beneficial long-term response to IFX in patients with CD in an adult Spanish cohort; meanwhile, Medrano et al. reported different results, associating this SNP with short-term non-response to IFX among patients with CD in an adult Italian cohort. Therefore, a deeper analysis that considers the population-related presence and effect of this SNP is needed.

The SNP rs763110 (CC + CT) of gene *FAS-L*, which is linked to apoptosis, was associated with responsiveness to IFX in patients with CD, regardless of whether the response was short-term or long-term [38].

Recently, the homozygous variants of gene *ATG16L1* (rs2241880 AA) and gene *PHATCR3* (rs6100556 TT) were found to be related to long-term non-response to IFX in a Spanish pediatric cohort with IBD [40].

**Table 1:** Polymorphisms associated with responsiveness among patients with Crohn’s disease, ulcerative colitis, or inflammatory bowel disease treated with IFX.

rs	Genotype	Disease	Rf or NRf: n [*p* (OR/HR; 95% CI)]	Long/Short Term	Response Criteria	Gene	Pathway	Reference
rs6100556	TT	CD and UC	NR: 340 [0.004 (aHR: 2.67; 1.37–5.23)]	Long	Inmunogenic, pharmacokinetic, and pharmacodynamic criteria	*PHACTR3*	Cell proliferation	[40]
rs2241880	AA	CD and UC	NR: 340 [0.006 (aHR: 0.37; 0.19–0.76)]	Long	Inmunogenic, pharmacokinetic, and pharmacodynamic criteria	*ATG16L1*	Autophagy	[40]
rs1813443	CC	CD	NR: 126 [0.002 (11.50; 2.50–52.84)]	Short	Luminal disease: HBI. Perianal disease: closure of fistulas or reduction of draining fistulas	*CNTN5*	Nervous system development; immunoglobulin superfamily	[39]
rs1816702	CC	CD	R: 132 [0.0049 (HR: 0.13; 0.02–0.99)]	Long	Clinical, biochemical, and endoscopic data or the need for abdominal surgery	*TLR2*	Pathogen recognition	[35]
rs1568885	TT	CD	NR: 126 [0.007 (21.37; 2.73–167.20)]	Short	Luminal disease: HBI. Perianal disease: closure of fistulas or reduction of draining fistulas	*CNTN5*	Nervous system development; immunoglobulin superfamily	[39]
rs1061624	AA or GA	CD	NR: 297 [0.015 (OR: 1.78; 1.09–2.90)]	Short	Luminal disease: HBI. Perianal disease: closure of fistulas or reduction of draining fistulas	*TNFRSF1B*	TNF-α pathway; inflammation	[41]
rs976881	AA and GA	CD	NR: 125 [ 0.014 (OR 3.30; 1.20–9.10)]	Short	No clinical response to IFX induction infusions	*TNFRSF1B*	TNF-α pathway; inflammation	[38]
rs3804099	TT	CD	R: 132 [0.023 (HR: 0.04; 0.18–0.88)]	Long	Clinical, biochemical, and endoscopic data or the need for abdominal surgery	*TLR2*	Pathogen recognition	[35]
rs1061624	AA or GA	CD	R: 132 [0.03 (HR: 0.04; 0.18–0.92]	Long	Clinical, biochemical, and endoscopic data or the need for abdominal surgery	*TNFRSF1B*	TNF-α pathway; inflammation	[35]
rs763110	CC or CT	CD	NR: 125 [0.041 (OR 4.00 (1.10–22.40)]	Short	No clinical response to IFX induction infusions	*FAS-L*	Apoptosis	[38]
rs396991	GG	CD and UC	NR: 76 (pediatric) [0.01 (OR: 6.58; 1.91–23.17)]	Long	PCDAI and PUCAI	*FCGR3A*	Antibody-dependent immune responses	[37]

NRf (allele/genotype frequency higher in nonresponsive patients), Rf (allele/genotype frequency higher in responsive patients), UC (ulcerative colitis), CD (Crohn’s disease), PCDAI (Weighted Pediatric Crohn’s Disease Activity Index), PUCAI (Pediatric Ulcerative Colitis Activity Index), Harvey–Bradshaw index (HBI), R (response), NR (non-response), HR (hazards ratio), OR (odds ratio), CI (confidence interval), IFX (infliximab), TNF (tumor necrosis factor).

### 3.3. Polymorphisms Associated with Responsiveness among Patients with Inflammatory Bowel Disease Treated with Adalimumab

Several SNPs have been connected with beneficial short- and long-term responsiveness to ADA in patients with CD included in a Slovenian cohort [42] and in a Spanish pediatric cohort [40] (Table 2).

For short-term response, a number of SNPs have been identified in genes coding for proinflammatory cytokines (*IL-27*; rs8049439 CT and TT), for T-cell activation (*PTGER4*; rs10512734 GG), apoptosis (*CASP9*; rs4645983 AA or AG), and autophagy (*ATG16L1*; rs10210302 CT and TT) [42]. However, the homozygous patients carrying the rs10210302 C allele in gene *ATG16L1* were linked to long-term non-response to ADA [42]. A 2023 study by Zapata-Cobo et al. found an association between the homozygous variant of the rs2241880 G allele in gen *ATG16L1* and long-term non-response in pediatric patients with IBD treated with ADA. In addition, the homozygous variant (rs4645983 GG) of gene *CASP9* was associated with short-term non-response to ADA [42].

For long-term response, the most investigated SNPs are related to DNA repair, chromatin organization, and transcription regulation (*C11orf30*; rs7927894 CC), controlling cell division cycles and regulating cyclin-dependent kinases (*CCNY*; rs12777960 CC), adaptive immunity (*NR12*; rs3814057 CC), and proinflammatory cytokines (*IL-13*; rs1295686 TT) [42].

### 3.4. Polymorphisms Associated with Responsiveness among Patients with Inflammatory Bowel Disease Treated with Both Infliximab and Adalimumab

The information from the studies has been summarized in Table 3. Related to the *TNF-α* gene, patients carrying the *TNF-α*-308 G, -238 G, or -857 C common alleles show better responses to TNF-α blockers than those with minor alleles, but only in White populations [43].

For long-term beneficial response among patients with UC, we found only the SNP rs11465996 CC + CG of gene *LY96*, which is associated with pathogen recognition [36].

For long-term non-response among patients with CD, SNPs have been identified in genes coding for anti-inflammatory cytokines (IL-10; rs1800872 CC), proinflammatory cytokines (IL-17; rs2275913 AA, IL-6; rs10499563 TT) [48], and in gene CDKAL1 (rs6098425 CC) [40], whose function remains unknown. One study associated the SNP rs2275913 AA or AG with non-response among a Danish cohort of patients with UC [36]. Another study found that the polymorphisms of genes ATGL16L1 (rs2241880 AA), PHATCR3 (rs6100556 TT), and IRF1-AS1 (rs2188962 CT + TT) were linked to long-term non-response to anti-TNF drugs in a Spanish pediatric cohort with UC [40].

In 2023, Zapata-Cobo et al. showed that the heterozygous variant (rs10508884 CC + CT) of gene *CXCL12* and the homozygous variants of genes *ATG16L1* (rs2241880 AA) and *PHATCR3* (rs6100556 TT) were associated with long-term non-response to anti-TNF biologics in a Spanish pediatric cohort with IBD. Carriers of the rs10508884C allele in *CXCL12* responded more poorly to anti-TNFs in the Kaplan–Meier univariate analysis (*p* = 0.049), although statistical significance was lost in a Cox regression analysis adjusted for sex, type of IBD, and type of anti-TNF drug (adjusted hazard ratio 0.309; 95% CI 0.076–1.268, *p* = 0.103).

The most investigated gene in IBD was HLA-DQA1*05, due to two variants in *HLA* genes (rs2097432 CC + CT and rs2395185 GG), which have been associated with a response to anti-TNF drugs. Thus, three SNPs were associated with non-response to both IFX and ADA among specific cohorts with IBD. The SNP rs2097432 was associated with non-response to anti-TNF drugs in a Spanish pediatric cohort with CD. Additionally, SNP rs2395185 has been linked to long-term non-response to anti-TNF drugs in a Spanish pediatric cohort with IBD (UC and CD) [46].

Another study [44] associated SNP rs2097432 (CC + CT) with long-term non-response to anti-TNF drugs in a cohort of European descent with CD. In 2016, Bek et al. performed a meta-analysis study that confirmed that nine polymorphisms in eight genes (*TLR2*, *CD14*, *LY96*, *TNF*, *TNFRSF1B*, *TNFAIP3*, *IL1RN*, and *IL17A)* were not associated with anti-TNF therapy in patients with IBD.

### 3.5. Polymorphisms Associated with Responsiveness among Patients with IBD Treated with Ustekinumab

Lastly, we found one genotype variant of the gene protein tyrosine phosphatase non-receptor type 2 (*PTPN2*; rs7234029 AG + GG) linked to non-response to UST among patients with CD in a German cohort. Moreover, another SNP rs2542151 for the same gene was studied but was not associated with responsiveness to UST [47].

## 4. Discussion

This review aimed to comprehensively summarize the most recent findings regarding the pharmacogenetics of the biologics anti-TNF-α (IFX and ADA), UST, and VDZ in IBD to shed light on the genetic variants that affect the clinical response to biologics. In this context, genome-wide association studies have identified SNPs that have a potential association with IBD pathogenesis [49]. A large proportion of these SNPs have been located in key cellular pathways, knowledge of which is of great importance in selecting the most effective therapeutic intervention to improve IBD management [50,51]. This is the case for anti-TNF-α, which is employed extensively for moderate to severe IBD, although not all patients show an optimal response to induction therapy, and, for others, the response fails over time for unknown reasons [52].

Several studies have demonstrated an association between SNPs and pharmacological responses to IBD therapies. As reported by Lauro R et al., *TNF-α* and *TNF receptor* (*TNFR*) polymorphisms (the A allele in *TNF-α*-308 and the G allele in *TNFRSF1A*) influence the response to anti-TNF-α therapy. Accordingly, one of the most important studies for evaluating the pharmacogenetic influence in IBD is the PANTS prospective study [14], an investigation that helped identify SNPs in the HLA-DQA1*05 allele, which is related to increased immunogenicity risk in patients with CD undergoing anti-TNF-α therapy [31,44]. Another SNP to consider is the non-synonymous polymorphism rs1061622, which has been shown to be associated with clinical CD phenotypes [53,54] and has been linked to functional alterations of the *TNFRSF1B* gene through mRNA transduction alteration [41]. It has been shown that rs1061622 participates in beneficial outcomes of both IFX induction and IFX maintenance therapy [38]. In addition, the atypical perinuclear antineutrophil cytoplasmic antibody (ANCA) serological marker is associated with the *TNFRSF1B* variant, and with IBD-related complications and non-response to anti-TNF [49].

Another SNP with implications in therapeutic response is rs1816702. Variant C in the homozygosis of rs1816702 in the *TLR2* gene (but not in the TT genotype) potentially predicts a better response to IFX [35]. The distribution of the minor rs976881 allele in the intronic sequence of *TNFRSF1B* differed between patients with a discontinuous IFX maintenance therapy and those who maintained clinical remission on IFX maintenance therapy. This important result clearly reflects that carrying the minor rs976881 allele can be associated with loss of response, and agrees with the previous finding that homozygosity for the minor allele can increase the risk of response failure more than minor allele heterozygotes. However, no association with primary non-response to IFX was observed for this SNP [38].

In the study of long-term response and non-response to IFX therapy, rs1813443, located in the intronic section of gene *CNTN5* [55], has been associated with clinical and biochemical responses to IFX in Greek patients with Crohn’s disease [39]. This result has similarly been observed for the TT genotype of the rs1568885 polymorphism, also located in gene *CNTN5*, showing a statistically significant relationship to partial response and resistance to IFX [39]. Another identified SNP with the TT genotype, rs763110, has a role in suppressing apoptosis by reducing the binding affinity of the *FasL* promoter for its target transcription factors [56] and has been associated with responsiveness to IFX.

In pediatric CD, the SNP rs8049439, related to cytokine *IL-27* and located in chromosome 16p11.2, has been specifically associated with the onset of IBD [57,58], although this SNP has also been associated with short-term response to ADA in adult patients with CD [42]. The role of this SNP and the related proinflammatory cytokine *IL-27* appears to be different in CD depending on the disease and treatment status. It is also important to mention rs10512734, in the 5p13.1 intergenic region, which includes the regulatory elements of the prostaglandin receptor *EP4* (*PTGER4*) gene. The beneficial association of this SNP to short-term ADA response in CD [42] is consistent with the information regarding gene *EP4*, and it can be a genuine susceptibility factor for CD in White populations [59].

The *caspase-9* (*CASP9*) gene is located at 1p36 and encodes a 416 amino acid cysteine protease, which initiates the activation of the caspase cascade and induces cell apoptosis. In IBD, classical therapeutic drugs such as sulfasalazine, mesalazine, and even IFX have been reported to induce lymphocyte apoptosis via caspase-3 activation. Therefore, polymorphisms of the *CASP9* gene, which is a potent upstream activator of *caspase-3*, could affect apoptosome formation and subsequently pro-caspase-3 cleavage [60]. The *CASP9* gene rs4645983 polymorphism has been associated with short-term non-response to ADA [42]. Therefore, *CASP9* variants could be the underlying factors influencing the imbalanced apoptosis in peripheral blood lymphocytes in patients with CD [2,3].

The *ATG16L1* gene is important in IBD because it is implicated in cellular autophagy processes and bacterial clearance in (innate) immune cells. Thus, SNPs in this gene could affect the bacterial composition of the gut of patients with IBD and immune recognition. In this context, the presence of rs10210302 correlates in CD with a beneficial response to ADA therapy [61,62]. SNP rs1295686, in the *IL-13* gene, is of biological interest given that it is implicated, as a functional enhancer, with the *TH2LCRR* promoter (T helper type 2 locus control region-associated RNA). This, in turn, is a recently identified long non-coding RNA whose presence in immune diseases such as asthma [63] could also indicate their potential role in IBD.

CXCL12 is a ubiquitous and constitutive chemokine that participates in important biological pathways, such as in the proliferation and migration of stem cells and the migration of T cells to the inflamed human intestinal mucosa [64,65]. A long-term study [40] found that rs10508884 in the *CXCL12* gene was associated with non-response in IBD (CD and UC), which is of interest due to the role of this chemokine in the migration of T cells to the inflamed gut [66].

In regard to SNPs related to UST response, the *PTPN2* gene has been implicated. PTPN2 is a classical cytoplasmic phosphatase, which is expressed ubiquitously. Interestingly, however, its highest level of expression is detected in lymphoid cells and can therefore be of importance in maintaining immune system homeostasis [67], with a crucial regulatory role in inflammasome activation and certain cytokine production during intestinal inflammation. The presence of rs7234029 with its significance in the clinical response to UST highlights the role of PTPN2 in IBD [67].

### Strength and Limitations

This systematic review is a comprehensive compilation of the current knowledge on genetic variants associated with response to biological treatment in patients with IBD. The potential candidate SNPs, as predictive biomarkers of response, are depicted in the tables and interpreted in the context of the existing scientific literature related to IBD. However, there are certain limitations that should be mentioned. Comparing the published articles is challenging because of the difficulty in reproducibility due to the high degree of heterogeneity among the included studies, which could be attributed to differences in the study design (pediatric or adult patients, and other demographic or clinical characteristics). Further prospective multicenter studies are needed to validate biological treatment response variations related to the described polymorphisms. Another aspect to consider is the fact that differences in genetic ancestry could introduce genetic variation, with the potential to alter the therapeutic efficacy of certain pharmacological therapies [68]. In this context, there is a need for studies that include participants from underrepresented ethnic groups to better understand the effects of pharmacogenetics on the biological therapeutic response.

In response to biological therapies in IBD, other influencing factors, such as environmental factors and their influence through epigenetic mechanisms, might also be considered, especially due to the heterogenous nature of IBD [69,70].

## 5. Conclusions

In the era of biological therapies for IBD, it is logical to determine which elements can modulate the therapeutic response to these therapies. As reviewed here, pharmacogenetics emerges as a key field for optimizing biological therapy in IBD. The genetic predisposition of patients with IBD to biologics, due to the presence of any of the described SNPs, can modulate the beneficial response to therapy and the disease’s progression. It is therefore important to perform a large-scale biological therapy analysis to power the results and to select the best candidate SNPs for implementation into routine clinical practice. To date, the most significant SNPs in this context are linked to immunity, such as cytokine production and immunorecognition, due to the immune-mediated nature of IBD.

## Figures and Tables

**Table 2:** Polymorphisms associated with responsiveness among patients with Crohn’s disease, ulcerative colitis, or inflammatory bowel disease treated with adalimumab.

rs	Genotype	Disease	Rf or NRf: n [*p* (OR/HR; 95% CI)]	Long/Short Term	Response Criteria	Gene	Pathway	Reference
rs10210302	CT or TT	CD	R: 102 [8.11 × 10^−4^ (OR: 9.44; 2.49–35.83)]	Short	CRP	*ATG16L1*	Autophagy	[42]
rs10210302	CC	CD	NR: 102 [4.05 × 10^−3^]	Long	N.D	*ATG16L1*	Autophagy	[42]
rs10512734	GG	CD	R: 102 [4.62 × 10^−3^]	Short	IBDQ	*PTGER4*	T-cell activation	[42]
rs8049439	CT or TT	CD	R: 102 [5.56 × 10^−3^]	Short	CRP	*IL-27*	Proinflammatory cytokine	[42]
rs7927894	CC	CD	R: 102 [5.83 × 10^−3^]	Long	CRP	*C11orf30*	DNA repair, chromatin organization, and regulation of transcription	[42]
rs1295686	TT	CD	R: 102 [6.07 × 10^−3^]	Long	IBDQ	*IL13*	Proinflammatory cytokine	[42]
rs12777960	CC	CD	R: 102 [6.69 × 10^−3^]	Long	CRP	*CCNY*	Control cell division cycles and regulate cyclin-dependent kinases	[42]
rs4645983	AA or AG	CD	R: 102 [6.91 × 10^−3^]	Short	IBDQ	*CASP9*	Apoptosis	[42]
rs4645983	GG	CD	NR: 102 [7.22 × 10^−3^]	Short	CRP	*CASP9*	Apoptosis	[42]
rs3814057	CC	CD	R: 102 [9.64 × 10^−3^]	Long	CRP	*NR12*	Adaptive immunity	[42]
rs2241880	GG	CD and UC	NR: 340 [0.026 (aHR: 2.39; 1.11–5.14)]	Long	Inmunogenic, pharmacokinetic, and pharmacodynamic criteria	*ATG16L1*	Autophagy	[40]

NRf (allele/genotype frequency higher in nonresponsive patients), Rf (allele/genotype frequency higher in responsive patients), CD (Crohn’s disease), UC (ulcerative colitis), CRP (C-reactive protein), IBDQ (Inflammatory Bowel Disease Questionnaire), R (response), NR (non-response), HR (hazards ratio), OR (odds ratio), CI (confidence interval).

**Table 3:** Polymorphisms associated with responsiveness among patients with Crohn’s disease, ulcerative colitis, or inflammatory bowel disease treated with infliximab and adalimumab.

rs	Genotype	Disease	Rf or NRf: n [*p* (OR/HR; 95% CI)]	Long/Short Term	Response Criteria	Gene	Pathway	Reference
rs2097432	CC or CT	CD	NR: 1240 [4.24 × 10^−13^ (HR: 1.70; 1.48–1.94)]/NR: 178 (independent cohort) [8.80 × 10^−4^ (HR: 1.69; 1.26–2.28)]	ND	Persistence	HLA-DQA1*05	Central role in the immune system by presenting peptides derived from extracellular proteins	[44]
rs116724455	CC	CD and UC	NR: 474 [4.79 × 10^−8^ (OR: 19.90; 4.57–86.70)]	ND	Continued use at the time of study enrolment without failure	*TNFSF4/18*	TNF-α pathway; inflammation	[45]
rs2228416	TT	CD and UC	NR: 474 [5.24 × 10^−6^ (OR: 5.25; 2.33–11.8)]	ND	Continued use at the time of study enrolment without failure	*PLIN2*	Lipid globule surface membrane material (development and maintenance of adipose tissue)	[45]
TNF-α 308	G Allele	CD	R: 667 [8.6 × 10^−5^ (OR: 2.01; 1.42–2.84)]	ND	CD: CDAI, HBI	*TNF-α*	TNF-α pathway; inflammation	[43]
rs6100556	TT	UC	NR: 340 [0.007; aHR: 2.95 (1.34–6.49)]	Long	Inmunogenic, pharmacokinetic, and pharmacodynamic criteria	*PHACTR3*	Cell proliferation	[40]
rs3804099	CC or CT	CD and UC	R: 738 [0.01 (ORadj: 1.80; 1.15–2.81)]	ND	Simple 3-step scale	*TLR2*	Pathogen recognition	[36]
TNF-α -857	C Allele	CD	R: 274 [0.013 (OR: 1.78; 1.13–2.80)]	ND	CD: Clinical outcome	*TNF-α*	TNF-α pathway; inflammation	[43]
TNF-α 238	G Allele	CD	R: 274 [0.016 (OR: 2.20; 1.16–4.15)]	ND	CD: HBI	*TNF-α*	TNF-α pathway; inflammation	[43]
rs2097432	CC o CT	CD	NR: 340 [0.019 (HR: 1.77; 1.10–2.85)]	Long	Clinical, biochemical, and endoscopic data or the need for abdominal surgery	HLA-DQA1*05	Central role in the immune system by presenting peptides derived from extracellular proteins	[46]
rs2430561	AA or AT	CD	R: 482 [0.02 (ORadj: 1.97; 1.13–3.42)]	ND	Simple 3-step scale	*IFNG*	Proinflammatory cytokine	[36]
rs4612666	CT or TT	CD and UC	NR: 1783 [0.02 (OR: 0.73; 0.57–0.95)]	ND	Simple 3-step scale	*NLRP3*	Regulation of inflammation, the immune response, and apoptosis	[4]
rs2241880	AA	CD and UC	NR: 340 [0.02 (aHR: 0.51; 0.29–0.90)]	Long	Inmunogenic, pharmacokinetic, and pharmacodynamic criteria	*ATG16L1*	Autophagy	[40]
rs11938228	AA or AC	UC	NR: 256 [0.02 (OR: 0.55; 0.33–0.92)]	ND	Simple 3-step scale	*TLR2*	Pathogen recognition	[4]
rs2188962	CT or TT	UC	NR: 340 [0.029 (3.24; aHR: 1.13–9.35)]	Long	Inmunogenic, pharmacokinetic, and pharmacodynamic criteria	*IRF1-AS1*	lncRNA	[40]
rs12343867	TT or TC	CD	R: 1069 [0.03 (OR: 1.35; 1.02–1.78)]	ND	Simple 3-step scale	*JAK2*	Proinflammatory cytokine	[4]
rs1554973	CC or CT	CD and UC	NR: 1783 [0.03 (OR: 0.80; 0.65–0.98)]	ND	Simple 3-step scale	*TLR4*	Pathogen recognition	[4]
rs6927172	GG or CG	UC	NR: 256 [0.03 (ORadj: 0.34; 0.13–0.90)]	ND	Simple 3-step scale	*TNFAIP3*	TNF-α pathway; inflammation	[36]
rs2395185	GG	CD and UC	NR: 340 [0.039 (HR: 0.60; 0.37–0.98)]	Long	Clinical, biochemical, and endoscopic data or the need for abdominal surgery	HLA-DQA1*05	It plays a central role in the immune system by presenting peptides derived from extracellular proteins	[46]
rs4149570	AA	CD	R: 1069 [0.04 (OR: 1.92; 1.02–3.60)]	ND	simple 3-step scale	*TNFRSF1A*	TNF-α pathway; inflammation	[4]
rs4848306	AA or AG	UC	R: 256 [0.04 (ORadj: 2.69; 1.04–6.94)]	ND	Simple 3-step scale	*IL1B*	Proinflammatory cytokine	[36]
rs4645983	TT	CD	R: 287 [0.04 (OR: 1.50; 1.34–1.68)]	ND	CDAI	*CASP9*	Apoptosis	[47]
rs352139	TT	CD	NR: 482 [0.04 (ORadj: 0.38; 0.16–0.94)]	ND	Simple 3-step scale	*TLR9*	Pathogen recognition	[36]
rs4696480	TT	UC	NR: 256 [0.04 (ORunadj: 0.47; 0.23–0.95)]	ND	Simple 3-step scale	*TLR2*	Pathogen recognition	[36]
rs5030728	AA	CD and UC	R: 1783 [0.04 (OR: 1.46; 1.01–2.11)]	ND	Simple 3-step scale	*TLR4*	Pathogen recognition	[4]
rs2569190	AA or AG	UC	NR: 256 [0.04 (ORunadj: 0.54; 0.30–0.98)]	ND	Simple 3-step scale	*CD14*	Pathogen recognition	[36]
rs696	GA or AA	CD and UC	R: 1783 [0.04 (OR: 1.25; 1.01–1.54)]	ND	Simple 3-step scale	*NFKBIA*	Pathogen recognition	[4]
rs1946518	GT or TT	CD and UC	R: 1783 [0.04 (OR: 1.24; 1.01–1.53)]	ND	Simple 3-step scale	*IL-18*	Proinflammatory cytokine	[4]
rs4251961	CC or CT	UC	NR: 256 [0.04 (ORadj: 0.42; 0.18–0.98)]	ND	Simple 3-step scale	*IL1RN*	Proinflammatory cytokine	[36]
rs6098425	CC	CD	NR: 340 [0.044 (aHR: 2.23; 1.02–4.88)]	Long	Inmunogenic, pharmacokineti, and pharmacodynamic criteria	*CDKAL1*	Unknown	[40]
rs187238	GG or GC	CD	R: 1069 [0.047 (OR: 1.35; 1.00–1.82)]	ND	Simple 3-step scale	*IL-18*	Proinflammatory cytokine	[4]
rs4251961	TC or CC	CD and UC	NR: 1783 [0.049 (OR: 0.81; 0.66–1.00)]	ND	Simple 3-step scale	*IL1RN*	Proinflammatory cytokine	[4]
rs10508884	CC or CT	CD and UC	NR: 340 [0.049 (HR: 0.27; 0.07–1.11)]	Long	Inmunogenic, pharmacokineti, and pharmacodynamic criteria	*CXCL12*	Proinflammatory cytokine	[40]
rs1800872	CC	CD	NR: 209 [<0.05 (HR: 4.75; 1.16–19.52)]	Long	CD: wPCDAI; UC: PUCAI	*IL-10*	Anti-inflammatory cytokine	[48]
rs2275913	AA	CD	NR: 209 [<0.05 (HR: 0.32; 0.11–0.92)]	Long	CD: wPCDAI; UC: PUCAI	*IL-17A*	Proinflammatory cytokine	[48]
rs11465996	CC or CG	UC	R: 309 [<0.05 (HR: 10.22; 1.85–56.50)]	Long	Simple 3-step scale	*LY96*	Pathogen recognition	[36]
rs2275913	AA or AG	UC	NR: 256 [0.05 (ORadj: 0.42; 0.18–1.00)]	ND	Simple 3-step scale	*IL17A*	Proinflammatory cytokine	[36]
rs10499563	TT	CD	NR: 209 [0.05 (HR: 0.21; 0.05–0.95)]	Long	CD: wPCDAI; UC: PUCAI	*IL-6*	Proinflammatory cytokine	[48]
rs6100556	TT	CD and UC	NR: 340 [aHR: 0.025 (1.93; 1.09–3.42)]	Long	Inmunogenic, pharmacokinetic, and pharmacodynamic criteria	*PHACTR3*	Cell proliferation	[40]

NRf (allele/genotype frequency higher in nonresponsive patients), Rf (allele/genotype frequency higher in responsive patients), ND (not defined), UC (ulcerative colitis), CD (Crohn’s disease), wPCDAI (Weighted Pediatric Crohn’s Disease Activity Index), PUCAI (Pediatric Ulcerative Colitis Activity Index). R (response), NR (non-response), HR (hazards ratio), OR (odds ratio), CI (confidence interval).

## Supplementary Materials

The following supporting information can be downloaded at https://www.mdpi.com/article/10.3390/ijms25073717/s1. Reference [71] is cited in the Supplementary Materials.

## List of Abbreviations

ADA	adalimumab
ATG16L1	autophagy-related 16 like 1
C11orf30	EMSY transcriptional repressor, BRCA2 interacting
CASP9	caspase 9
CCNY	cyclin Y
CD	Crohn’s disease
CD14	CD14 molecule
CDAI	Crohn’s Disease Activity Index
CDKAL1	CDK5 regulatory subunit associated protein 1 like 1
CI	confidence interval
CNTN5	contactin 5
CRP	C-reactive protein
CXCL12	C-X-C motif chemokine ligand 12
FAS-L	Fas ligand
FCGR3A	Fc gamma receptor IIIa
HAUS6	HAUS augmin-like complex subunit 6
HBI	Harvey–Bradshaw Index
HLA-DQA1*05	major histocompatibility complex, class II, DQ alpha 1
IBD	inflammatory bowel disease
IBDQ	Inflammatory Bowel Disease Questionnaire
IFNG	interferon gamma
IFX	infliximab
IL-	interleukin (1B, 6, 10, 12, 13, 17A, 18, 23 or 27)
IL1RN	interleukin 1 receptor antagonist
IRF1-AS1 (CARINH)	colitis-associated IRF1 antisense regulator of intestinal homeostasis
JAK2	Janus kinase 2
LY96	lymphocyte antigen 96
NFKBIA	NFKB inhibitor Alpha
NLRP3	NLR family pyrin domain containing 3
NR12	greater CFTR locus negative regulatory element NR12
OR	odds ratio
PHACTR3	phosphatase and actin regulator 3
PLIN2	perilipin 2
PTGER4	prostaglandin E receptor 4
PTPN2	protein tyrosine phosphatase non-receptor type 2
PUCAI	Pediatric Ulcerative Colitis Activity Index
SNP	single nucleotide polymorphism
Th17	lymphocyte T helper 17
TH2LCRR	T helper type 2 locus control region-associated RNA
TLR	toll-like receptor (2, 4 or 9)
TNF	tumor necrosis factor
TNFAIP3	TNF alpha-induced protein 3
TNFR	TNF receptor (type 1 or 2)
TNFRSF1	TNF receptor superfamily member 1 (A or B)
TNFSF	TNF superfamily member (4 or 18)
UC	ulcerative colitis
UST	ustekinumab
wPCDAI	Weighted Pediatric Crohn’s Disease Activity Index
