# Supplementary material for: Genetic Variants Associated with Biological Treatment Response in Inflammatory Bowel Disease: A Systematic Review
Source: Int J Mol Sci. 2024 Mar 27;25(7):3717. doi: 10.3390/ijms25073717 (PMC11012229; doi:10.3390/ijms25073717)
Supplement: Supplementary file 1 [file ijms-25-03717-s001.zip › Supp. Table S1. Paper search strategy.pdf]

Supplementary Table S1. Search strategy with full list of search terms.

Alternative names for medicine		Alternative names for genetic variation		Alternative names for illness
Anti tnf OR antitnf OR anti-tnf OR tnf blocker OR tnf blockers OR tnf inhibitor OR tnf inhibitors OR anti-tnf treatment OR Infliximab OR Remicade OR Humira OR adalimumab OR simponi OR golimumab OR tumor necrosis factor blocker OR tumor necrosis factor inhibitor OR tumor necrosis factor blockers OR tumor necrosis factor inhibitors OR tumor necrosis factor-alpha antagonist OR tumor necrosis factor antagonist OR tumor necrosis factor-alpha antagonists OR tumor necrosis factor antagonists OR Ustekinumab OR Anti-IL12/23 OR IL12/23 blockers OR Anti IL12/23 OR IL12/23 inhibitors OR IL12/23 antagonist OR IL12/23 anatgonists OR Vedolizumab OR anti-α4β7 OR α4β7 inhibitors OR α4β7 blockers OR α4β7 antagonists OR α4β7 antagonist OR biological drugs OR biological drugs treatment OR biological drugs treatments OR biological drugs therapy OR biological drugs therapies	AND	Polymorphisms OR polymorphism OR genetic variants OR genetic variant OR polymorphism OR DNA polymorphisms OR DNA polymorphism OR gene polymorphism OR gene polymorphisms OR genetic polymorphism OR genetic polymorphisms OR Single nucleotide polymorphism OR Single nucleotide polymorphisms OR SNP OR SNPs OR Polymorphism single nucleotide OR Genetic variation OR genome-wide association study OR genome-wide association OR Genome-Wide Association Studies OR Genome Wide Association Scan OR Genome Wide Association Studies OR GWA Study OR GWA Studies OR GWAS OR Whole Genome Association Analysis OR Whole Genome Association Study OR Genome Wide Association Analysis OR Genome Wide Association Study OR Genome Wide Association Studies OR Genetic association study OR Genetic association studies OR Genetic association	AND	inflammatory bowel disease OR crohn disease OR crohn's disease OR ulcerative colitis
