# Supplementary material for: Genetic Variants Associated with Biological Treatment Response in Inflammatory Bowel Disease: A Systematic Review
Source: Int J Mol Sci. 2024 Mar 27;25(7):3717. doi: 10.3390/ijms25073717 (PMC11012229; doi:10.3390/ijms25073717)
Supplement: Supplementary file 1 [file ijms-25-03717-s001.zip › Suppl. Table S2. Extended response criteria.pdf]

Supplementary Table S2. Extended response criteria.

A. **Infliximab:**

SNPs	Response criteria	Bibliographic reference
rs1816702, rs3804099, rs1061624	Clinical, biochemical and endoscopic data or the need for abdominal surgery related to CD progression.	Salvador-Martín et al., 2019
rs1061624, rs763110	No clinical response to IFX induction infusions at weeks 0, 2, and 6 and consequently followed by IFX discontinuation	Steenholdt et al., 2012
rs1813443, rs1568885	Luminal disease: Harvey– Bradshaw index (HBI) at the beginning and 10 weeks after the first IFX dose. Partial response was defined as a decrease in the HBI of more than 3 points and absence of concomitant corticosteroids. Remission was defined as a final HBI < 4 and absence of concomitant corticosteroids.<br>Perianal disease: at week 10 after the first IFX dose. Remission was defined as the complete closure of all fistulas and partial response as a reduction (P50%) in the number of draining fistulas. Patients who received IFX for both luminal and fistulizing disease and who achieved remission of any type (in the luminal or in the fistulizing disease) that justified maintenance treatment with IFX were considered as responders. All patients who did not achieve partial response or remission after the three IFX induction doses were considered as primary nonresponders.	Medrano et al., 2014
rs396991	Pediatric Crohn's Disease Activity Index (PCDAI) and Pediatric Ulcerative Colitis Activity Index (PUCAI) for patients with CD and UC, respectively, at the end of induction and during maintenance phase (at 22 and 52 weeks). Disease was considered in remission if indexes were less than or equal to 10.	Curci et al,. 2021
rs2241880, rs6100556	Failure of biologic treatment was defined as a change in the anti-TNF drug used due to loss of response according to immunogenic, pharmacokinetic, and pharmacodynamic criteria established by the clinician	Zapata-Cobo et al., 2023

B. **Adalimumab**:

SNPs	Response criteria	Bibliographic reference
rs10210302, rs10512734, rs8049439, rs7927894, rs12777960, rs3814057, rs1295686, rs4645983	IBD questionnaire (IBDQ) and biological response using CRP values, both for 4, 12, 20 and 30 weeks after treatment. Response was defined as an increase in IBDQ for more than 22 points (ΔIBDQ > 22) or as an IBDQ value higher than 170 points. Biological response was defined as a decrease of CRP to normal values (<3 mg/l) or drop in CRP levels by more than 25%.	Koder et al., 2015

rs2241880	Failure of biologic treatment was defined as a change in the anti-TNF drug used due to loss of response according to immunogenic, pharmacokinetic, and pharmacodynamic criteria established by the clinician	Zapata-Cobo et al., 2023

C. Infliximab and adalimumab:

**SNPs**	**Response criteria**	**Bibliographic reference**
rs4149570, rs187238, rs12343867, rs11938228, rs696, rs5030728, rs696, rs1946518, rs1554973, rs4612666, rs4251961	3-step scale reflected the best response within 26 weeks after initiation. CD or UC with luminal disease were categorised as having: (A) no response, meaning no improvement or worsening of symptoms; (B) partial response, meaning some improvement of symptoms or reduction of steroid dose without worsening of symptoms; (C) response, meaning absence or almost absence of all clinical symptoms without increasing the steroid dose. Fistulising CD were categorised as having: (A) no response, meaning no improvement or worsening of symptoms; (B) partial response, meaning reduced secretion or discomfort from fistulas or closure of one or some of the fistulas; (C) response, meaning closure of all fistulas evaluated by thumb pressure or no secretion.	Bank et al., 2014
rs2430561, rs352139, rs4848306, rs4696480, rs2569190, rs6927172, rs4251961, rs2275913, rs3804099	Treatment efficacy using the simple three-step scale reflected the maximum response within 22 weeks after initiation	Bank et al., 2019
rs2097432	Persistence: defined as the length of time from initiation of anti-TNF to exit from the study due to study failure	Sazonovs et al., 2020
rs1800872, rs2275913, rs10499563	CD patients: remission was defined as a wPCDAI<12.5 points after completing the induction period and response as >17.5-point change in the initial wPCDAI (weighted Pediatric Crohn Disease Activity Index) value without achieving remission. UC patients: clinical remission was defined as PUCAI (Pediatric Ulcerative Colitis Activity Index) <10 points, and clinically significant response was defined by a PUCAI change of at least 20 points, or entering remission	Salvador-Martín et al., 2020
rs116724455, rs2228416	regarding primary outcome of response to anti-TNF therapy, 'Yes' was defined as being treated with an anti-TNF agent, infliximab or adalimumab, for the first time, with continued use at the time of study enrolment without failure to any biologics, and 'No' was defined as neither infliximab nor adalimumab being effective after treatment.	Wang et al., 2019
rs10508884, rs2241880, rs6100556, rs6100556, rs2188962, rs2241880, rs6098425	Failure of biologic treatment was defined as a change in the anti-TNF drug used due to loss of response according to immunogenic, pharmacokinetic, and pharmacodynamic criteria established by the clinician	Zapata-Cobo et al., 2023
TNF-α 238, TNF-α -857, TNF-α 308	Evaluation of response: Harvey– Bradshaw index (HBI) and clinical outcomes	Song et al,. 2015

D. Ustekinumab:

**SNPs**	**Response criteria**	**Bibliographic reference**
rs7234029, rs2542151	Nonresponse to medical therapy was defined by a switch to a different medical therapy within six months of treatment initiation due to primary lack or secondary loss of efficacy or need for surgery. Response to medical therapy was defined by the patient remaining on the same medical therapy through month 6 from treatment initiation	Hoffmann et al., 2021
